# Characterization and Computational Engineering of Structural Elements Controlling Gas Permeability in PIP2;1 Aquaporins

**DOI:** 10.1002/jcc.70377

**Published:** 2026-04-25

**Authors:** Ahmad Raeisi Najafi, Paween Mahinthichaichan, Fraser J. Moss, Ardeschir Vahedi‐Faridi, Walter F. Boron, Emad Tajkhorshid

**Affiliations:** ^1^ Theoretical and Computational Biophysics Group, NIH Resource for Macromolecular Modeling and Visualization, Beckman Institute for Advanced Science and Technology, Department of Biochemistry, and Center for Biophysics and Quantitative Biology University of Illinois Urbana‐Champaign Urbana Illinois USA; ^2^ Department of Mechanical Engineering and Mechanics Drexel University Philadelphia Pennsylvania USA; ^3^ Department of Physiology and Biophysics Case Western Reserve University School of Medicine Cleveland Ohio USA; ^4^ Department of Pathology Case Western Reserve University School of Medicine Cleveland Ohio USA; ^5^ Department of Medicine Case Western Reserve University School of Medicine Cleveland Ohio USA; ^6^ Department of Biochemistry Case Western Reserve University School of Medicine Cleveland Ohio USA

## Abstract

Aquaporins (AQPs) are classical water channels that also conduct small gas molecules such as $O_{2}$ and ${\mathrm{CO}}_{2}$ across the membrane. The hydrophobic central pore, located at the fourfold symmetry axis of an AQP tetrameric architecture, has been proposed to constitute the most optimal pathway for gas transport, although monomeric water pores can also contribute somewhat to permeation of less hydrophobic species. Here, we report a comparative molecular dynamics (MD) study of gas permeability in a plant AQP and a mammalian AQP1, taking advantage of complementary computational protocols including flooding simulations, umbrella sampling, and implicit ligand sampling. PIP2;1 AQPs, present in plants, are experimentally reported to have lower gas permeability than AQP1, which is present both in plants and animals. Using the spinach PIP2;1 (SoPIP2;1) and bovine AQP1 (bAQP1) as the models, the study unravels the specific structural features controlling the permeability of the central pore to gases. In SoPIP2;1, residue Trp79, which is highly conserved in the plant PIP2;1 family and lines directly the central pore, forms a major constriction region and the main barrier against gas permeation. Notably, the occluding conformation of the four Trp79 residues from the four monomers is stabilized by another conserved residue, Phe207 in the central pore. Sequence and structural comparisons show that both of these residues are replaced by less bulky residues in AQP1, for example, by Leu56 and Ala179, respectively, in bAQP1. The role of Phe207 residues in hindering gas permeation through SoPIP2;1 is confirmed by in silico alanine substitution, which reveals its effect on the local constriction produced by Trp79 residues. Conversely, by mutating Leu56 to tryptophan and Ala179 to phenylalanine in bAQP1, we engineer the protein to a less permeable gas channel.

## Graphical abstract

Bulky residues constrict the central pore of WT SoPIP2;1, generating high energy barriers for both O_2_ and CO_2_ and preventing gas permeation.

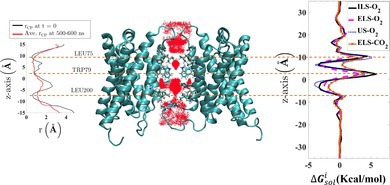

## 1 Introduction

The permeability of cellular membranes to physiologically significant gaseous molecules, such as ${\mathrm{CO}}_{2}$ and $O_{2}$, is key to the proper functioning of many living organisms. Respiration and photosynthesis are two major examples of such functions that heavily rely on the transport of gas molecules across membranes. In the past century, our understanding of membrane permeability to gas molecules has evolved substantially [1, 2]. In two independent studies at the beginning of the last century, Meyer and Overton concluded that cell membranes are highly permeable to all small hydrophobic molecules [3, 4]. However, many later studies have shown that the permeability of biological membranes to certain species can be orders of magnitude lower than predicted by the Meyer–Overton model [3, 4, 5, 6, 7, 8].

Cellular membranes are generally highly heterogeneous, containing different amounts of integral and peripheral membrane proteins, as well as varying degrees of diverse lipids, for example, phospholipids and sterols [9, 10]. As a result, their gas permeability can differ significantly from that of single‐lipid, synthetic lipid bilayers commonly used in experiments [11, 12, 13] and often in simulations. In this context, specialized gas transport proteins have been proposed to contribute to and enhance gas permeability of cellular membranes [1], whereas other proteins have been demonstrated to reduce the apparent gas permeability of artificial membranes [14]. In particular, a number of studies have suggested that aquaporins (AQPs), which are primarily known as water channels, can contribute to gas transport across the cellular membranes [14, 15, 16, 17, 18, 19, 20].

In the past decade, there has been considerable debate over the physiological significance of AQPs in the transport of gas molecules across cellular membranes. Some studies support the notion that ${\mathrm{CO}}_{2}$ permeability of membranes is increased by AQPs [11], while others oppose this idea [5, 7, 8]. These contrasting hypotheses were discussed in cross‐talk articles co‐authored by key proponents and opponents of the gas channel hypothesis [15, 21, 22]. The initial evidence indicating that AQPs may enhance the plasma membrane ${\mathrm{CO}}_{2}$ permeability $\left(P_{M,{\mathrm{CO}}_{2}}\right)$ derives from investigations in which heterologously expressed human AQP1 (hAQP1) was shown to significantly increase $P_{M,{\mathrm{CO}}_{2}}$ in Xenopus oocyte. This hAQP1‐mediated increase in $P_{M,{\mathrm{CO}}_{2}}$ is sensitive to inhibition by both *p*‐chloromercuribenzene sulfonate (pCMBS) and 4,4′‐diisothiocyanatostilbene‐2,2′‐disulfonate (DIDS) [20, 23].

The permeability of plant AQPs to ${\mathrm{CO}}_{2}$ has also been studied. The plasma membrane intrinsic proteins (PIP) subfamily of AQPs constitutes one of the most investigated plant AQP families for their permeability properties. On the basis of sequence homology, the PIP subfamily is categorized into the PIP1 and PIP2 subfamilies [24]. The PIP1 subfamily includes five isoforms (PIP1;1 to PIP1;5), whereas the PIP2 subfamily has eight members (PIP2;1 to PIP2;8) [25, 26]. In general, PIP1s demonstrate low or no water permeability, while PIP2s act as efficient water channels [25, 27, 28, 29, 30]. Mammalian AQP0, AQP1, AQP4‐M23, AQP5, AQP6, and AQP9 all exhibit ${\mathrm{CO}}_{2}$ permeability [31], whereas the number of PIPs reported to contribute to $P_{M,{\mathrm{CO}}_{2}}$ is limited [32], and the results of investigations examining this property are inconsistent [32]. As suggested by these studies, PIPs can be grouped into three functional classes: permeable to water only, permeable to ${\mathrm{CO}}_{2}$ only, or permeable to both water and ${\mathrm{CO}}_{2}$.

Some, but not all, members of both PIP1 and PIP2 families appear to be able to facilitate ${\mathrm{CO}}_{2}$ transport [32]. The tobacco PIP1, NtAQP1, showed ${\mathrm{CO}}_{2}$ transport activity in *Xenopus laevis* oocytes [33]. In another study, Otto et al. [34] reported that heterologously expressed NtAQP1 in yeast cells increased the rate of ${\mathrm{CO}}_{2}$‐induced acidification. Two works from the Kaldenhoff group indicate that NtAQP1 functions as a ${\mathrm{CO}}_{2}$ channel, primarily within the inner chloroplast membrane, and that elevated expression of NtAQP1 enhances the plant's ${\mathrm{CO}}_{2}$ transport capacity, whereas reduced expression diminishes ${\mathrm{CO}}_{2}$ permeability and overall mesophyll ${\mathrm{CO}}_{2}$ conductance [35, 36].

The *Arabidopsis thaliana* PIP1AQP, AtPIP1;2, also functions as a physiologically relevant ${\mathrm{CO}}_{2}$ channel, the knockout of which was shown to cause a reduction in both whole‐leaf mesophyll ${\mathrm{CO}}_{2}$ conductance and the direct ${\mathrm{CO}}_{2}$ uptake rate of isolated mesophyll cells [37, 38], confirming that AtPIP1;2 expression limits the pathway for ${\mathrm{CO}}_{2}$ diffusion across the cell membrane.

While AtPIP1;2's role as a physiologically relevant ${\mathrm{CO}}_{2}$ transport facilitator is well established by both whole‐leaf conductance measurements [37] and cellular uptake assays [38], the ${\mathrm{CO}}_{2}$ permeability of the PIP2 subfamily remains a subject of debate. PIP2s are canonically characterized as water channels, with several studies suggesting they lack any significant ${\mathrm{CO}}_{2}$ transport activity. For example, NtPIP2;1 did not enhance ${\mathrm{CO}}_{2}$ permeability when heterologously expressed in yeast [34]. Similarly, Heckwolf et al. [37] demonstrated that unlike for AtPIP1;2 knockout, AtPIP2;3 knockout showed no deviation from the wild type (WT) mesophyll ${\mathrm{CO}}_{2}$ conductance, suggesting that AtPIP2;3 is not a major ${\mathrm{CO}}_{2}$ conduction facilitator in *Arabidopsis*.

In contrast to these findings, Hanba et al. [39] reported that overexpression of the barley AQP HvPIP2;1 in rice plants significantly enhanced both photosynthetic rate and mesophyll ${\mathrm{CO}}_{2}$ conductance; indirectly indicating that HvPIP2;1 possesses a ${\mathrm{CO}}_{2}$‐related function. Similarly, when heterologously expressed in Xenopus oocytes the barley PIP2 isoforms HvPIP2;1, HvPIP2;2, HvPIP2;3, and HvPIP2;5 facilitate ${\mathrm{CO}}_{2}$ transport [40]. However, although sharing high sequence homology with HvPIP2;3, in HvPIP2;4 the presence of a methionine at position 254 instead of the highly conserved isoleucine 254 among most other barley PIP2 isoforms renders HvPIP2;4 ${\mathrm{CO}}_{2}$‐impermeable [40]. Nonetheless, the possession of Ile254 is not the sole determinant of ${\mathrm{CO}}_{2}$ permeability.

**FIGURE 1:**
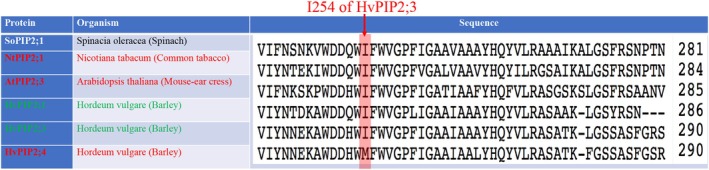
Multiple sequence alignment of some barley PIP2s against other plant PIP2s. PIP2s in red are reported to be impermeable to ${\mathrm{CO}}_{2}$, while PIP2s in green have been reported to facilitate ${\mathrm{CO}}_{2}$ transport across the membrane. The shaded region shows the Ile residue at position 254 of HvPIP2;3, which is conserved in all PIP2s presented in the figure, except in HvPIP2;4, which possesses a Met at this position.

Figure 1 shows that both NtPIP2;1 and AtPIP2;3 possess the conserved Ile254, yet neither facilitates ${\mathrm{CO}}_{2}$ transport when expressed in yeast or plants [34, 37]. Additional structural determinants must therefore also contribute to PIP gas permeability. Besides the monomeric pore, several studies have proposed a crucial role for parallel pathways including the hydrophobic central pore—formed at the tetrameric interface—in conducting ${\mathrm{CO}}_{2}$ [20, 31, 34, 41, 42, 43, 44, 45, 46]. The gas permeation properties of PIP central pores are poorly characterized compared to the water pores within each PIP protomer.

The present study employs an extensive set of simulations, including explicit ligand sampling (ELS) simulations (600 ns equilibrium simulations with multiple copies of ${\mathrm{CO}}_{2}$ or $O_{2}$ molecules, also known as flooding simulations) to study directly diffusion of gas molecules, as well as implicit ligand sampling (ILS) [47] and umbrella sampling (US) simulations [48, 49] to characterize the energetics of $O_{2}$ and ${\mathrm{CO}}_{2}$ partitioning in and passage through the central pore of a plant AQP, namely, Spinach PIP2;1 (SoPIP2;1), for which high‐resolution structures are available.

Our results show that the central pore of WT SoPIP2;1 is largely impermeable to $O_{2}$ and ${\mathrm{CO}}_{2}$. ELS, ILS, and US simulations reveal two major energy barriers against the permeation of $O_{2}$ and ${\mathrm{CO}}_{2}$ through this pathway. Analyzing the structure and radius profile of the central pore, we determine that these energy barriers are associated with the two constriction regions formed by bulky side chains. Guided by these results, we performed an in silico mutagenesis, replacing these bulky residues with smaller amino acids (e.g., Ala), to remove the constrictions and convert the central pore of SoPIP2;1 to a more favorable pathway for gas permeation. Furthermore, to validate our characterization of central pore barriers in SoPIP2;1, we computationally engineered a double mutant of bAQP1 (L56W/A179F) with an impermeable central pore to $O_{2}$ and ${\mathrm{CO}}_{2}$. Modeling and free energy calculations confirmed that this double mutant introduces a significant energy barrier in bAQP1, analogous to the blockage caused by Trp79 in SoPIP2;1.

## 2 Materials and Methods

### 2.1 Simulation Systems

The simulation systems for SoPIP2;1 and bAQP1 were independently constructed using their respective crystal structures. The structures of SoPIP2;1 were resolved in its closed (water pore) conformation at 2.1 Å (PDB: 1Z98), and in its open conformation at 3.9 Å (PDB: 2B5F) [50]. bAQP1 was resolved at 2.2 Å (PDB: 1J4N) [51]. The tetrameric form of each protein was constructed from the monomeric form by VMD [52] applying the transformation matrices provided in each PDB file. The 3.9‐Å structure of SoPIP2;1 was already provided as a tetramer, in which bound water molecules were modeled using the coordinates of its higher‐resolution 2.1 Å structure [50]. Each protein was embedded in a lipid bilayer, after predicting the membrane topology by the OPM (Orientations of Proteins in Membranes) server [53, 54]. CHARMM‐GUI [55] was used to insert the proteins in the membrane. POPC lipids were used for bAQP1, and POPE lipids were used for SoPIP2;1. The membrane‐embedded protein was then solvated with TIP3P water and ionized to 0.2 M NaCl using the Solvate and Autoionize plugins of VMD [52]. The final, solvated, membrane‐embedded models of SoPIP2;1 and bAQP1 comprised 113,506 and 105,650 atoms, respectively.

### 2.2 Simulation Protocol

In preparation for the flooding simulations and ILS analysis, a series of relaxation molecular dynamics (MD) simulations was performed, consisting of the following steps: (1) 0.5‐ns simulation with restraints (k=1 kcal/mol/Å^2^) applied to heavy atoms of the protein; (2) 0.5‐ns simulation with only backbone atoms of the protein restrained (k=1 kcal/mol/Å^2^); (3) 2‐ns simulation with only $C_{\alpha }$ atoms of the protein restrained; and, (4) 20‐ns unrestrained relaxation. Energy minimization (1,000 steps) was performed at the beginning of Steps 1–3 using the conjugate gradient algorithm. These steps were completed in the absence of $O_{2}$ or ${\mathrm{CO}}_{2}$.

All simulations were performed using NAMD2 [56, 57] with a time step of 2 fs and the CHARMM36 force field parameter set [58, 59, 60]. Periodic boundary conditions (PBC) were used throughout the simulations. All covalent bonds involving hydrogen atoms were kept rigid using the SHAKE algorithm [61]. The particle mesh Ewald (PME) method [62] with a grid density of 1/Å^3^ was used to evaluate long‐range electrostatic interactions without truncation. The cutoff for van der Waals interactions was set to 12 Å with a switching distance of 10 Å. The temperature was maintained at 310 K by Langevin dynamics [63] with a damping coefficient γ of 1/ps. The modified Nosé‐Hoover Langevin piston method [63, 64], in which Langevin dynamics is used to control fluctuations in the barostat, was used with a piston period of 200 fs, to maintain the pressure at 1 atm.

### 2.3 Implicit Ligand Sampling (ILS)

The 20‐ns unrestrained relaxation simulations (Step 4 of the protocol above) were extended for another 80 ns, adding up to a total of 100 ns. These simulations were then used to probe implicitly partitioning of $O_{2}$ molecules using the ILS method [47]. The method follows the assumption that small, hydrophobic ligands (here, $O_{2}$ molecules) only weakly interact with the protein, and therefore their presence would not perturb the protein structure and dynamics. Therefore, sampling the protein dynamics in the absence of the ligand can provide a close description of how it would behave when the ligand is explicitly present. ILS places the ligand at all possible positions (defined as a grid covering the region of interest) and calculates ligand‐protein interaction energies ($E_{i}$) at each position over an ensemble of protein conformations, also taking into account orientational entropy of the ligand by placement of the ligand in several different orientations [47]. Accordingly, the method can estimate a 3D free energy map for the ligand at position i, $\Delta G_{i}$.

$$\Delta G_{i}=-\mathrm{RT}\ln\frac{p_{i}}{p_{0}}=-\mathrm{RT}\ln\frac{<e^{-E_{i}/RT}>}{p_{0}}$$

where $p_{0}$ (in vacuum) = 1 and $p_{i}$ is the probability of moving a gas molecule from vacuum to position i. It is important to note that ILS may produce inaccurate results for polar molecules, such as ${\mathrm{CO}}_{2}$ which has significant atomic partial charges of +0.6 e for the C atom and −0.3 e for the O atoms. For $O_{2}$, which has negligible partial charges of ±0.02 e on each atom, ILS is expected to accurately predict free energy landscape. As such, here we rely mainly on the US results for ${\mathrm{CO}}_{2}$ permeation energetics.

Because ILS treats ligand orientations implicitly and assumes weak, non‐specific interactions between the ligand and the protein environment, it is less reliable for nonpolar ligands that nevertheless contain polar bonds, such as ${\mathrm{CO}}_{2}$. For these species, the free‐energy landscape depends strongly on orientation‐specific electrostatic interactions, which are not explicitly captured by ILS. Therefore, while ILS provides accurate results for nonpolar ligands such as $O_{2}$, the ${\mathrm{CO}}_{2}$ profiles should be interpreted with caution. In this work, we rely primarily on ELS (see Section 2.4) for quantitative characterization of ${\mathrm{CO}}_{2}$ permeation energetics.

In our study, the last 80 ns of the equilibrium simulations was used for the ILS analysis. The trajectories were divided into eight 10‐ns fragments, (i.e., t= 20–30 ns, t= 30–40 ns, …), each comprising 1000 frames. $O_{2}$ molecules were sampled in a $10\times 10\times 75\;{Å}^{3}$ grid with a spacing of 1 Å, covering the entire central pore of bAQP1 or SoPIP2;1 and extending into the aqueous solutions on either side. Ten orientations of $O_{2}$ were sampled in each subgrid, which contained 3×3×3 interaction sites. The solvation free energy of $O_{2}$ in water ($\Delta G_{\mathrm{sol}}$) was used as the reference for calculating the partitioning free energy of $O_{2}$ at each position i with respect to the solution ($\Delta G_{\mathrm{sol}}^{i}$).

$$\Delta G_{\mathrm{sol}}^{i}=\Delta G_{i}-\Delta G_{\mathrm{sol}},$$

$\Delta G_{\mathrm{sol}}$ was independently calculated over a $30\times 30\times 30\;{Å}^{3}$ part of the NaCl solution ILS, which yielded a $\Delta G_{\mathrm{sol}}$ value of 2.1 kcal/mol.

### 2.4 Explicit Ligand Sampling (ELS)—Flooding Simulations

A new set of simulations, in which explicit copies of $O_{2}$ or ${\mathrm{CO}}_{2}$ were added following the initial 20‐ns, unrestrained relaxation, was carried out to probe directly the diffusion of gas molecules, as well as their partitioning in and permeation through bAQP1 and SoPIP2;1. To ensure broad coverage of binding sites and gas pathways, and to capture permeation events within the limited timescale of a few hundred nanoseconds, a relatively high gas concentration (∼500 mM initial aqueous concentration comprising 125 molecules) was introduced. Each simulation lasted 600 ns. The force field parameters of $O_{2}$ and ${\mathrm{CO}}_{2}$ are available in the CHARMM36 force field [60].

Using the equilibrium fractions of the simulations, the apparent partitioning free energy of a gas molecule with respect to the solution ($\Delta G_{\mathrm{sol}}^{i}$) was directly estimated as:

$$\Delta G_{\mathrm{sol}}^{i}=-RT\ln\frac{p_{i}}{p_{\mathrm{sol}}},$$

where $p_{i}$ and $p_{\mathrm{sol}}$ represent time‐averaged probabilities of a gas species at position i and in the aqueous solution, respectively. Thus, $\Delta G_{\mathrm{sol}}^{i}$ is the transfer free energy of a gas species from the aqueous solution into a defined region (i).

Convergence of the ELS simulations was assessed by monitoring the temporal stability of gas‐occupancy distributions and the corresponding free‐energy profiles along the pore axis. Free‐energy profiles computed at intermediate simulation times were compared with those obtained from the full 600‐ns trajectories and showed no significant drift in either the locations or the heights of the major energy barriers. In addition, the resulting occupancy distributions exhibited the expected symmetry of the tetrameric central pore and displayed smooth *z*‐density profiles without nonphysical fluctuations. Agreement between ELS‐derived barriers and those obtained independently from ILS and US further supported that the simulations achieved sufficient sampling.

### 2.5 Umbrella Sampling (US) Simulations

To calculate the energetics of inserting $O_{2}$ and ${\mathrm{CO}}_{2}$ molecules in the central pore of SoPIP2;1, US simulations [48] were performed. The US simulations comprised ninety‐one 0.5‐Å windows, spanning from z=−20 Å to z=25 Å. The reference point (z=0) was defined as the center of mass of the AQP tetramer. To set up the initial ligand coordinates to each umbrella/window, two sets of steered MD simulations [65, 66] were performed with a gas molecule pulled from z=0 towards either the periplasmic solution (z=25 Å) or the cytoplasmic solution (z=−20 Å) at a velocity of 5 Å/ns with a force constant of $k=20\;\text{kcal}/\mathrm{mol}/{Å}^{2}$. In the US simulations, a harmonic potential with $k=10\;\text{kcal}/\mathrm{mol}/{Å}^{2}$ was applied to confine the gas molecule to the center of each window; each window was simulated for 3 ns.

To construct the ΔG profiles, the last 2.5 ns of the US trajectories were analyzed using the weighted histogram analysis method (WHAM) [49, 67], as implemented in LOOS [68], with a 0.25 Å histogram bin. Insertion ΔG values of the ligand (gas molecule) in individual bins ($\Delta G_{i}$) were subtracted by ΔG in the bulk solution ($\Delta G_{\mathrm{sol}}$), yielding partitioning free energies ($\Delta G_{\mathrm{sol}}^{i}$).

## 3 Results and Discussion

### 3.1 ${\mathrm{CO}}_{2}$ and $O_{2}$ Permeability of the Central Pore of SoPIP2;1

**FIGURE 2:**
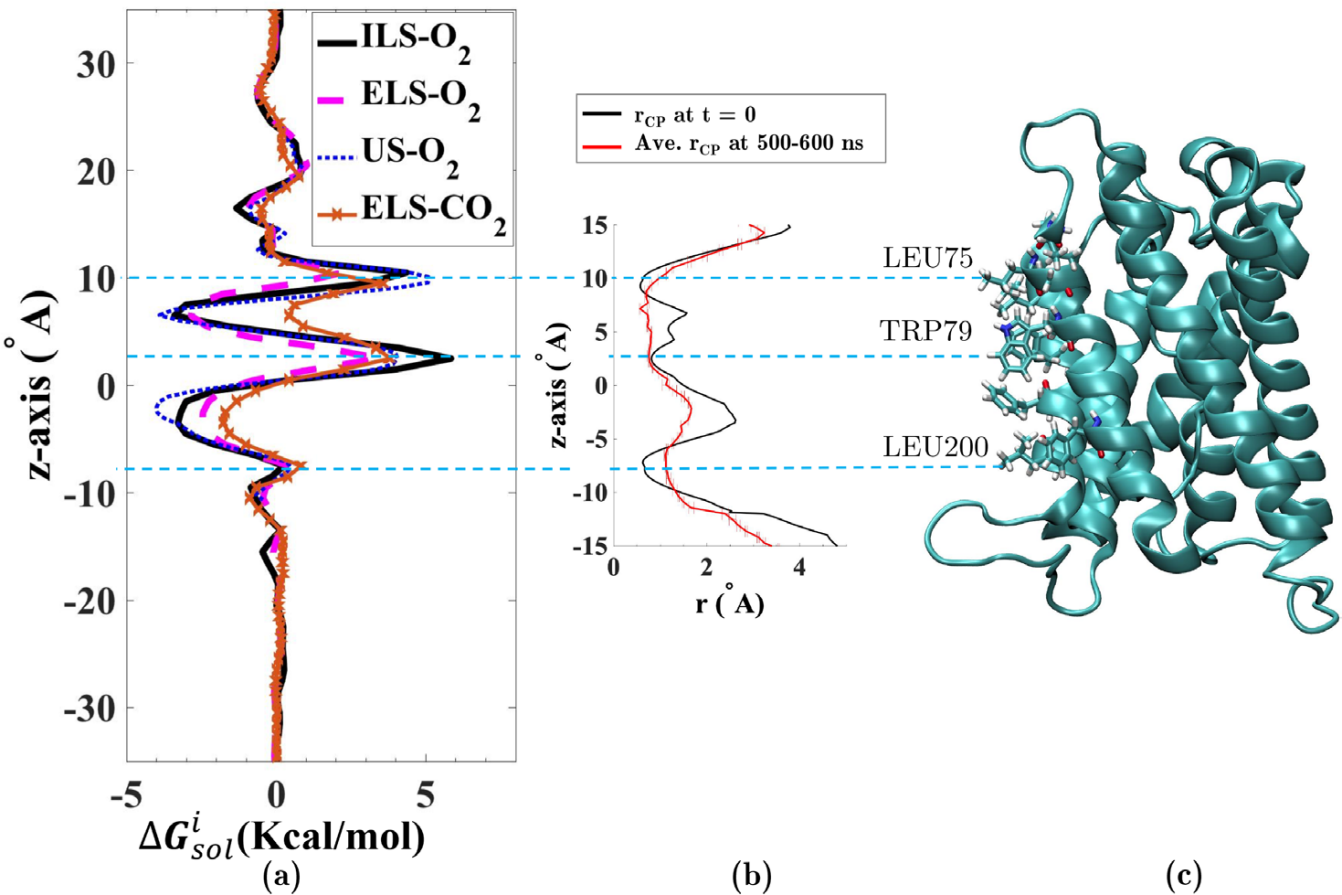
(a) 1D free‐energy profiles associated with $O_{2}$ and ${\mathrm{CO}}_{2}$ diffusion through the central pore of SoPIP2;1, calculated using ILS, ELS (600 ns), and US. (b) The central pore radius profile of SoPIP2;1 calculated by HOLE [69]. The pore radius at t = 0 is plotted in black, and the average radius during the last 100 ns of the simulation (from 500 to 600 ns) is shown in red. (c) One monomer of SoPIP2;1, aligned with the radius profile and the free energy profiles, where three hydrophobic residues Leu75, Trp79, and Leu200 projecting into the central pore are highlighted.

The gas permeability through the central pore of SoPIP2;1 was probed by ILS, flooding (ELS), and US simulations. As shown in Figure 2, the free energy profiles associated with $O_{2}$ permeation through the central pore, calculated with these three complementary approaches, are in very good agreement. Two energy wells are predicted for $O_{2}$: one at z=−2.1 Å with a ΔG between −4.0 and −2.2 kcal/mol, and another at z=+6.5 Å with a ΔG between −3.9 and −2.8 kcal/mol (Figure 2). The free energy profiles also reveal two major energy barriers against $O_{2}$ permeation: one at z=+2.5 Å with a ΔG between +3.5 to +5.8 kcal/mol, and another at z=+10.5 Å, with a ΔG between +2.2 and +5.1 kcal/mol. The ELS simulations predict very similar locations of the energy wells and barriers for ${\mathrm{CO}}_{2}$ diffusion through the central pore (Figure 2). These simulations indicate two energy barriers of +3.8 and +3.6 kcal/mol at z=+2.5 and z=+9.5 Å, respectively, for ${\mathrm{CO}}_{2}$ permeation. These results contrast with previously reported free energy profiles for gas permeation through the central pore of other AQPs, such as AQP1 and AQP4, which predict nearly barrier‐free and favorable gas conduction pathways [42, 46, 70]. Using ILS and ELS simulations, Wang et al. reported that free energy profiles associated with ${\mathrm{CO}}_{2}$ and $O_{2}$ permeation through the central pore of bAQP1 exhibit an energy well of −0.4 to −1.7 kcal/mol in the middle [42]. Based on these results, they suggested that the hydrophobic central pore of bAQP1 is permeable to ${\mathrm{CO}}_{2}$ and $O_{2}$. A similar conclusion was reported by Hub and de Groot [46].

In order to characterize the difference, we analyzed the shape and size of the central pore, using HOLE [69]. As shown in Figure 2b, the energy barriers in the central pore are located at and correspond to the narrowest regions of the central pore, marked by three bulky residues, Leu75, Trp79, and Leu200. The radius profile indicates constriction regions demarcated by minimum radii of r=0.6 Å at z=−7.2 Å (Leu200), r=0.8 Å at z=2.8 Å (Trp79), and r=0.6 Å at z=9.5 Å (Leu75). The results highlight the critical role of central pore size in governing gas conductance in plant AQPs.

**FIGURE 3:**
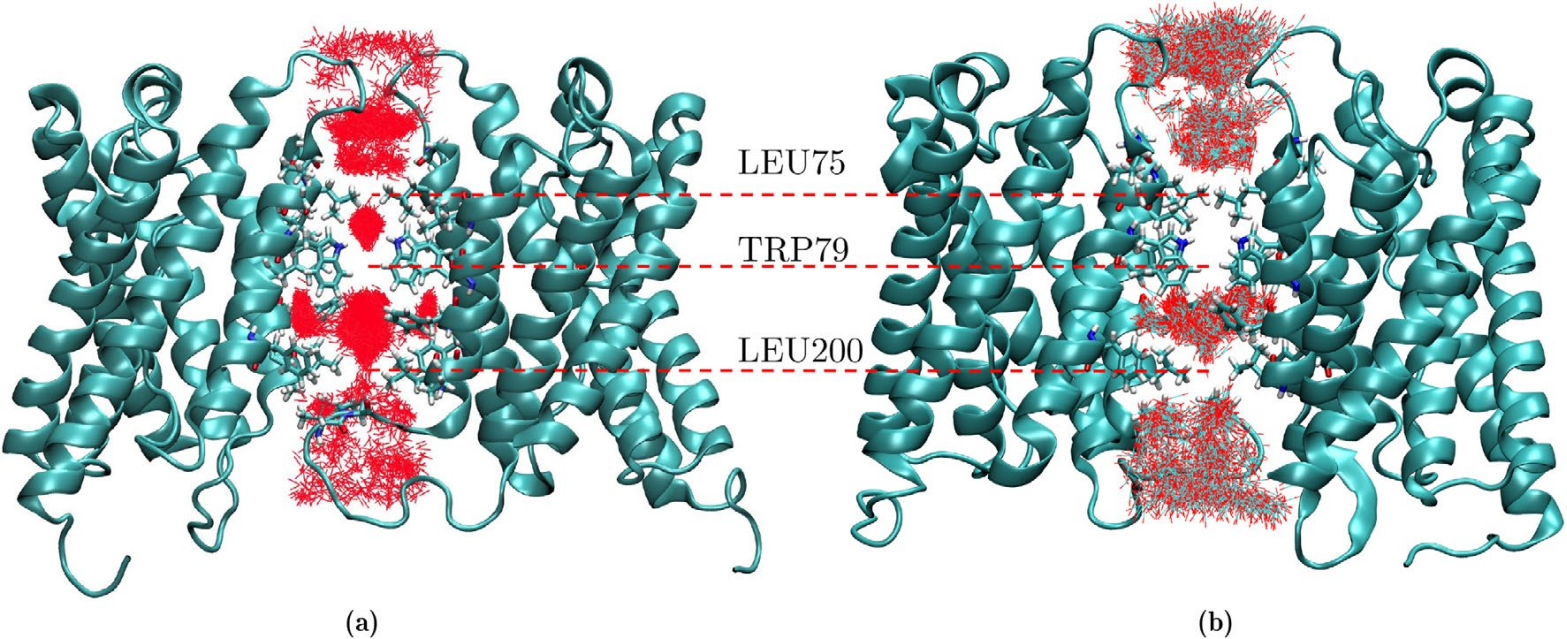
Trajectory of $O_{2}$ (a) and ${\mathrm{CO}}_{2}$ (b) molecules in the central pore during the first 200 ns of the simulations. These MD results predict that $O_{2}$ and ${\mathrm{CO}}_{2}$ molecules can barely pass through the high barrier regions associated with the conserved residue Trp79 in the central pore of SoPIP2;1.

Figure 3a,b shows an overlay of trajectory frames for $O_{2}$ and ${\mathrm{CO}}_{2}$ in the central pore during the first 200 ns of the ELS simulations, respectively. As shown, both $O_{2}$ and ${\mathrm{CO}}_{2}$ are barely able to pass through the segments of the central pore with high‐energy barriers. If three residues associated with high barriers (Leu75, Trp79, and Leu200) are mutated to smaller residues, for example, alanines, the barriers are expected to be eliminated and a favorable pathway for permeation of gas molecules through the protein would arise. The results of the simulations of such mutants are presented in Section 3.2.

### 3.2 In Silico Mutagenesis of Residues Lining the Central Pore

**FIGURE 4:**
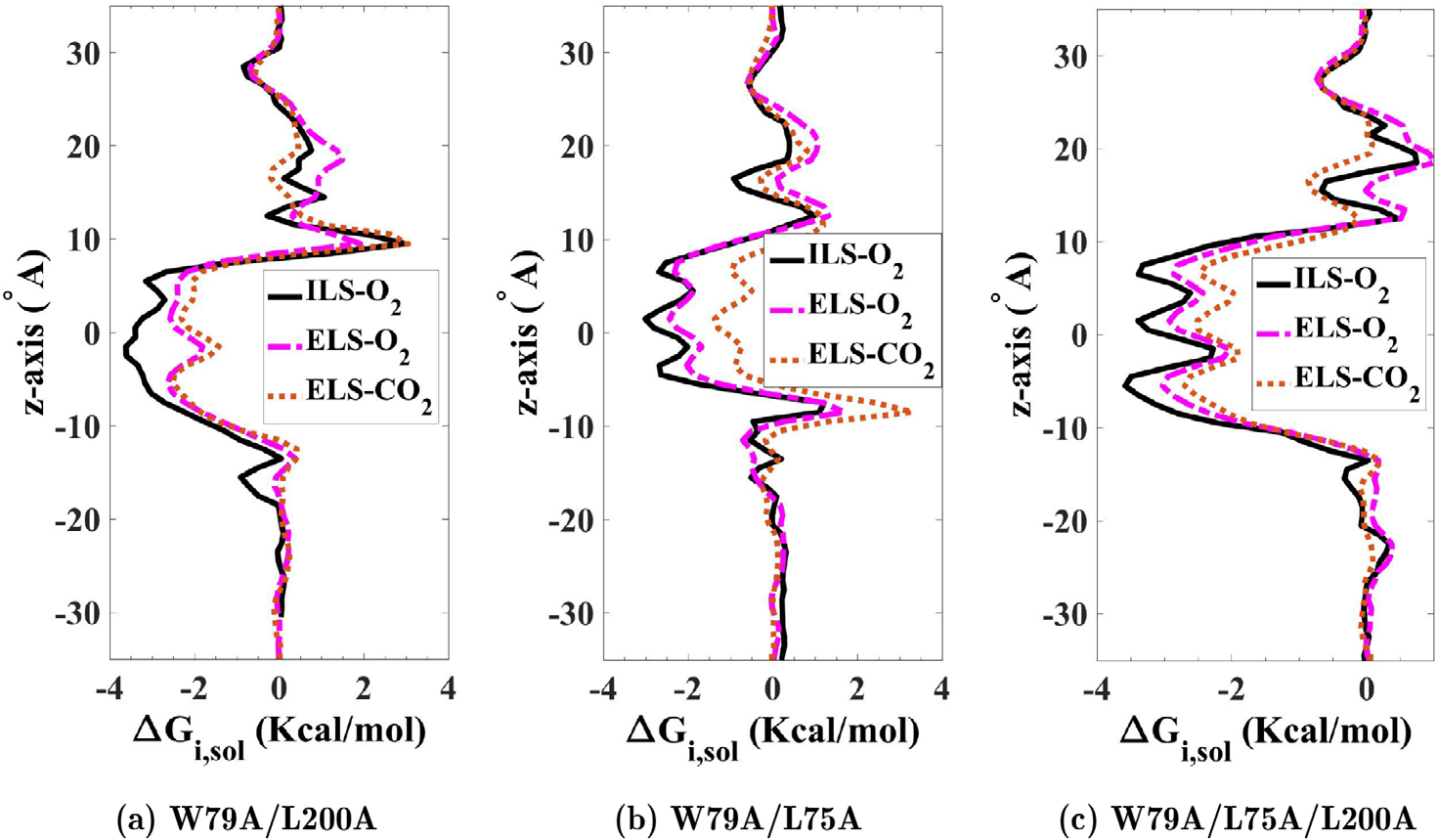
Energy profiles for in silico SoPIP2;1 mutants: (a) W79A/L200A double mutant, (b) W79A/L75A double mutant, and (c) W79A/L75A/L200A triple mutant. The free‐energy profiles of $O_{2}$ and ${\mathrm{CO}}_{2}$ calculated from ELS and ILS simulations indicate that both the double (W79A/L200A and W79A/L75A) and triple (W79A/L75A/L200A) mutations eliminate the free energy barriers associated with bulky residues, Leu75, Trp79, and Leu200, against $O_{2}$ and ${\mathrm{CO}}_{2}$ translocation.

Three different mutants associated with the central pore were computationally modeled to assess their impact on the free energy profiles and the diffusion of $O_{2}$ and ${\mathrm{CO}}_{2}$. The mutants include the W79A/L200A and W79A/L75A double mutants, and the W79A/L75A/L200A triple mutant. To compute free energy profiles for $O_{2}$ and ${\mathrm{CO}}_{2}$ across the central pore, we performed explicit gas diffusion simulations for 200 ns. For $O_{2}$, the results of flooding simulations were further validated by independent free energy calculations using ILS. As illustrated in Figure 4, all three mutants eliminated the energy barriers to $O_{2}$ and ${\mathrm{CO}}_{2}$ translocation along the central pore.

**FIGURE 5:**
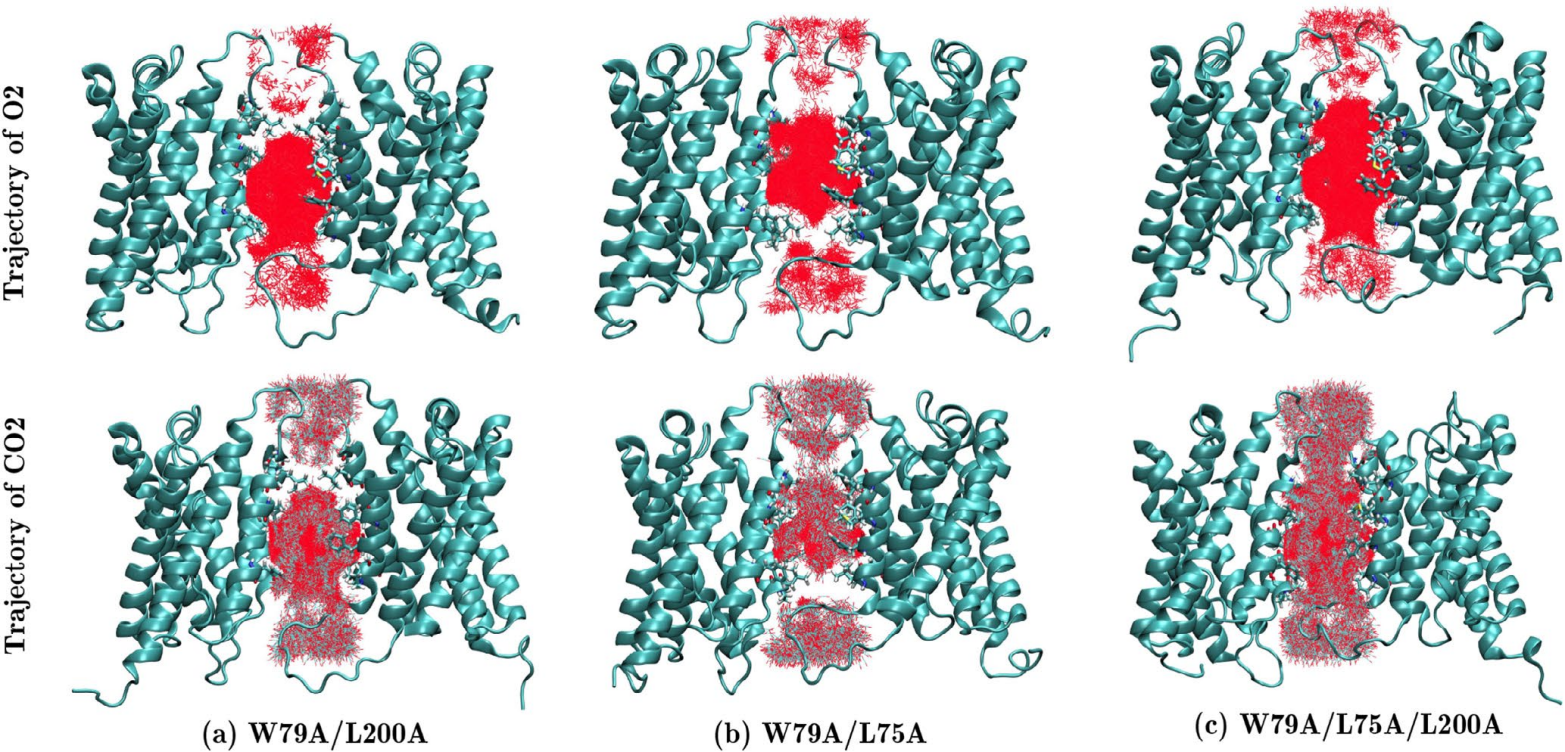
Trajectory of $O_{2}$ (top row) and ${\mathrm{CO}}_{2}$ (bottom row) through the central pore of (a) W79A/L200A double mutant, (b) W79A/L75A double mutant, and (c) W79A/L75A/L200A triple mutant of SoPIP2;1. Simulation time was 200 ns.

**FIGURE 6:**
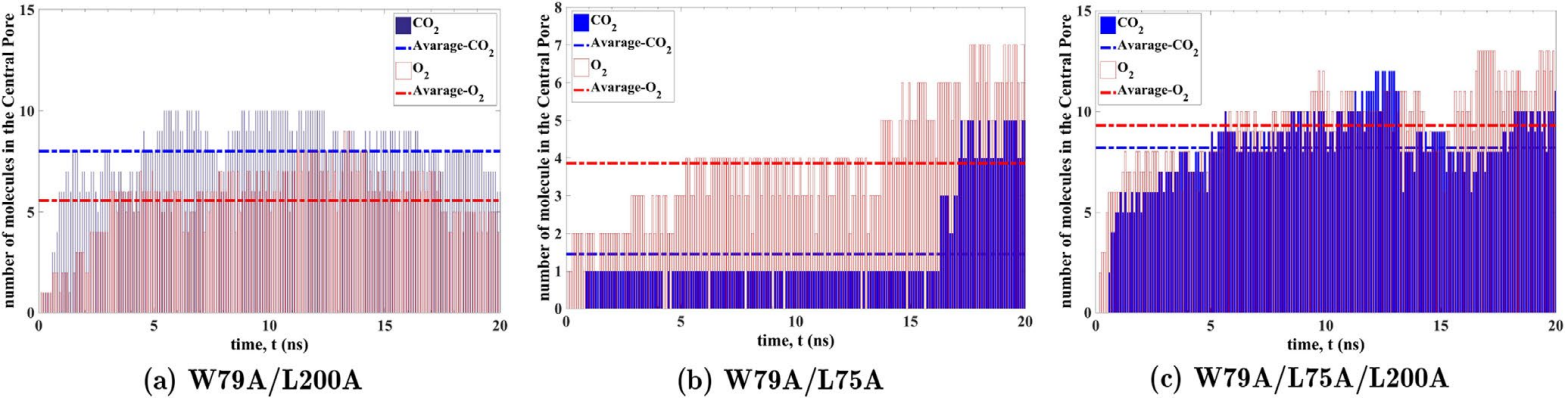
The number of $O_{2}$ and ${\mathrm{CO}}_{2}$ molecules that enter the central pore of (a) W79A/L200A double mutant, (b) W79A/L75A double mutant, and (c) W79A/L75A/L200A triple mutant of SoPIP2;1 during the first 20 ns of simulation.

The trajectories of $O_{2}$ and ${\mathrm{CO}}_{2}$ within the central pore, obtained from 200 ns flooding simulations, are shown in Figure 5. The results indicate that the permeability of $O_{2}$ and ${\mathrm{CO}}_{2}$ through the central pore increases in these mutants. In fact, in all three mutants, the central pore acts as a gas reservoir filled with $O_{2}$ and ${\mathrm{CO}}_{2}$. The number of gas molecules entering the pore during the first 20 ns of ELS simulations for each mutant is plotted in Figure 6.

### 3.3 Engineering a Gas‐Impermeable Central Pore in bAQP1

**FIGURE 7:**
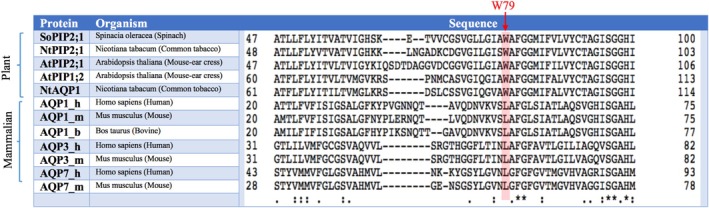
Partial sequence alignment of some plant AQPs against homologous mammalian AQPs. Shaded region highlights a conserved tryptophan lining the central pore in plant AQPs, which corresponds to a conserved leucine in mammalian AQPs.

Previous MD simulations showed that the central pore of bAQP1 is permeable to $O_{2}$ and ${\mathrm{CO}}_{2}$ [42, 46]. These findings are supported by experimental evidence [11, 71], which demonstrated that AQP1 channels can increase the ${\mathrm{CO}}_{2}$ permeability of membranes. Here, we aimed to design a bAQP1 central pore with reduced gas permeability using insights gained from the central pore architecture of SoPIP2;1. Figure 7 presents a partial sequence alignment of selected plant AQPs and mammalian AQPs. A conserved tryptophan (W) in plant AQPs is replaced with a different conserved residue, a leucine (L), in mammalian AQPs. Based on this observation, we hypothesized that substituting the native residue Leu56 in bAQP1 with tryptophan could reduce its gas permeability.

Therefore, to mimic the low gas permeability of plant SoPIP2;1, we first constructed an in silico single mutant of bAQP1, L56W, in which the conserved leucine at position 56 was replaced by tryptophan. This substitution was intended to introduce steric hindrance from the bulkier side chain within the central pore, thereby creating a barrier to gas transport and reducing the gas permeability of bAQP1. We then employed flooding simulations and ILS calculations to evaluate the gas permeability of the central pore in this mutant.

**FIGURE 8:**
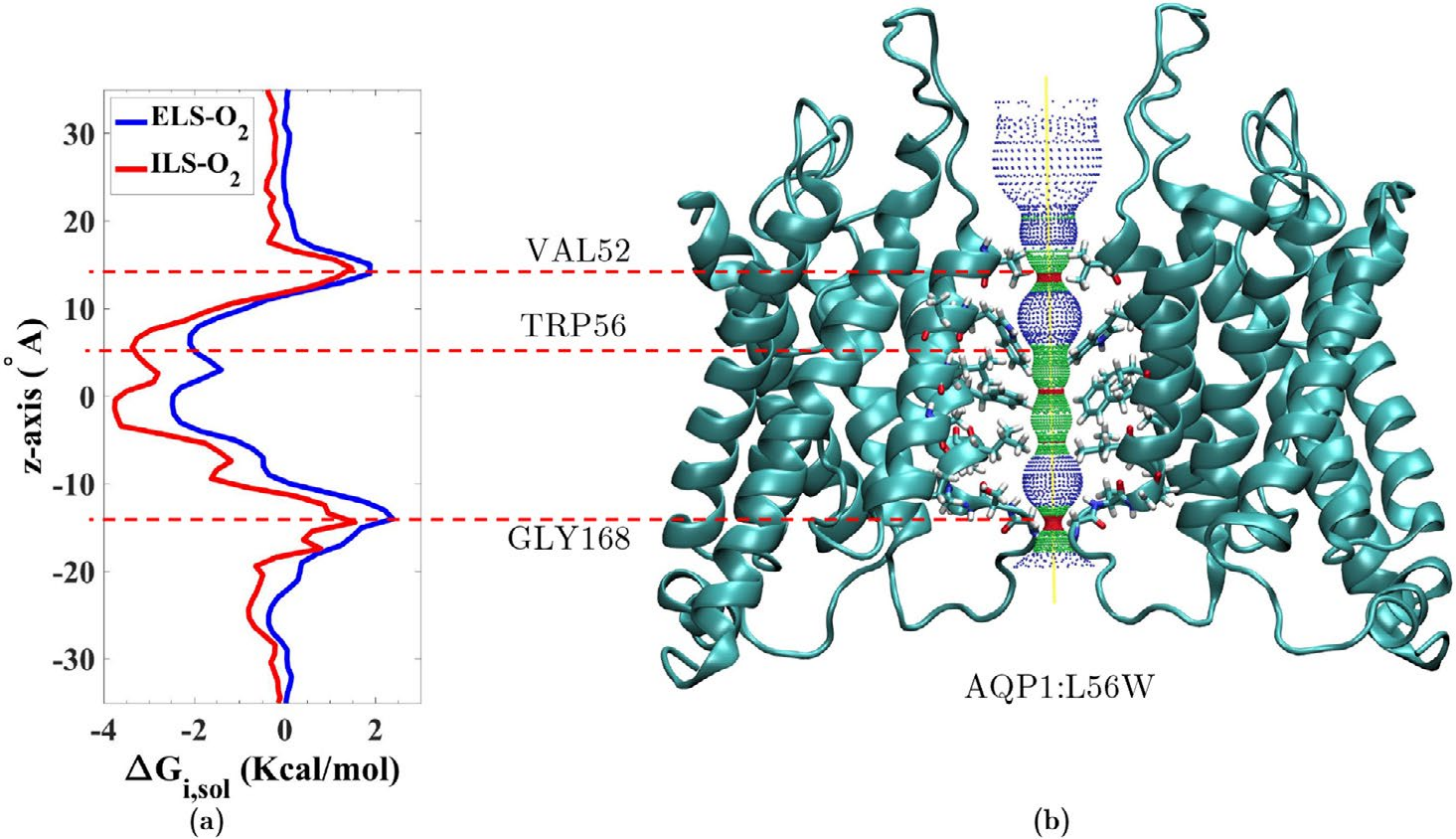
(a) 1D free‐energy profiles of $O_{2}$ transport in bAQP1:L56W do not show a major energy barrier in the central pore. (b) Molecular structure of the mutant bAQP1:L56W in ribbon representation and aligned with the energy profile in (a), along with the corresponding radius profile (colored mesh) calculated with HOLE [69]. Only two opposing monomers are shown to allow a clear view of the central pore. Some of the pore‐lining residues including Val52, Trp56, and Gly168 are explicitly shown. The narrowest parts of the pore are specified with red color in the radius profile.

The one‐dimensional free‐energy profiles of $O_{2}$ transport along the central pore are plotted in Figure 8. The results, surprisingly, show that this mutation has only a modest effect on the free energy barriers of $O_{2}$ translocation. To investigate the underlying reason, we examined the positional configuration of native Trp79 in WT SoPIP2;1 and Trp56 in the mutated bAQP1:L56W during the simulations.

**FIGURE 9:**
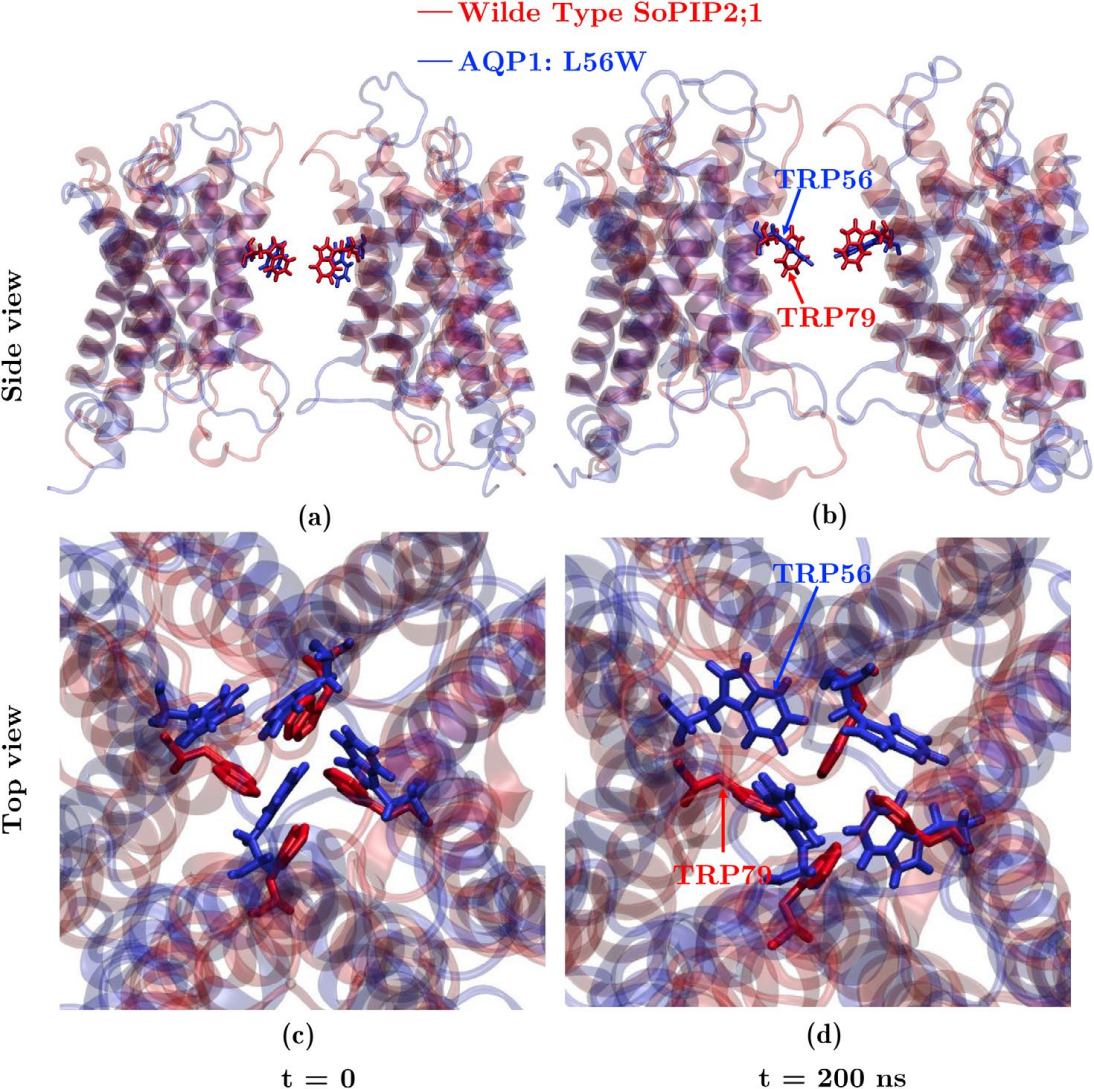
Structural overlay of the WT SoPIP2;1 (red) and mutated AQP1:L56W (blue) in side view (top row) and top view (bottom row) at t=0 (a, c) and t=200 ns (b, d). Trp56 of the bAQP1:L56W mutant undergoes large motion and changes its configuration, whereas native Trp79 of SoPIP2;1 stays in the same configuration during 200 ns simulation.

The structural overlay of WT SoPIP2;1 and mutated bAQP1:L56W is presented in Figure 9, where native Trp79 (SoPIP2;1) and Trp56 (mutated bAQP1) are displayed at both the beginning and end of the 200 ns simulations. While Trp79 in WT SoPIP2;1 exhibits only minor configurational changes between t=0 and t=200 ns, the introduced Trp56 in bAQP1:L56W undergoes a substantial rearrangement during the simulations, largely clearing the transport pathway in the central pore.

**FIGURE 10:**
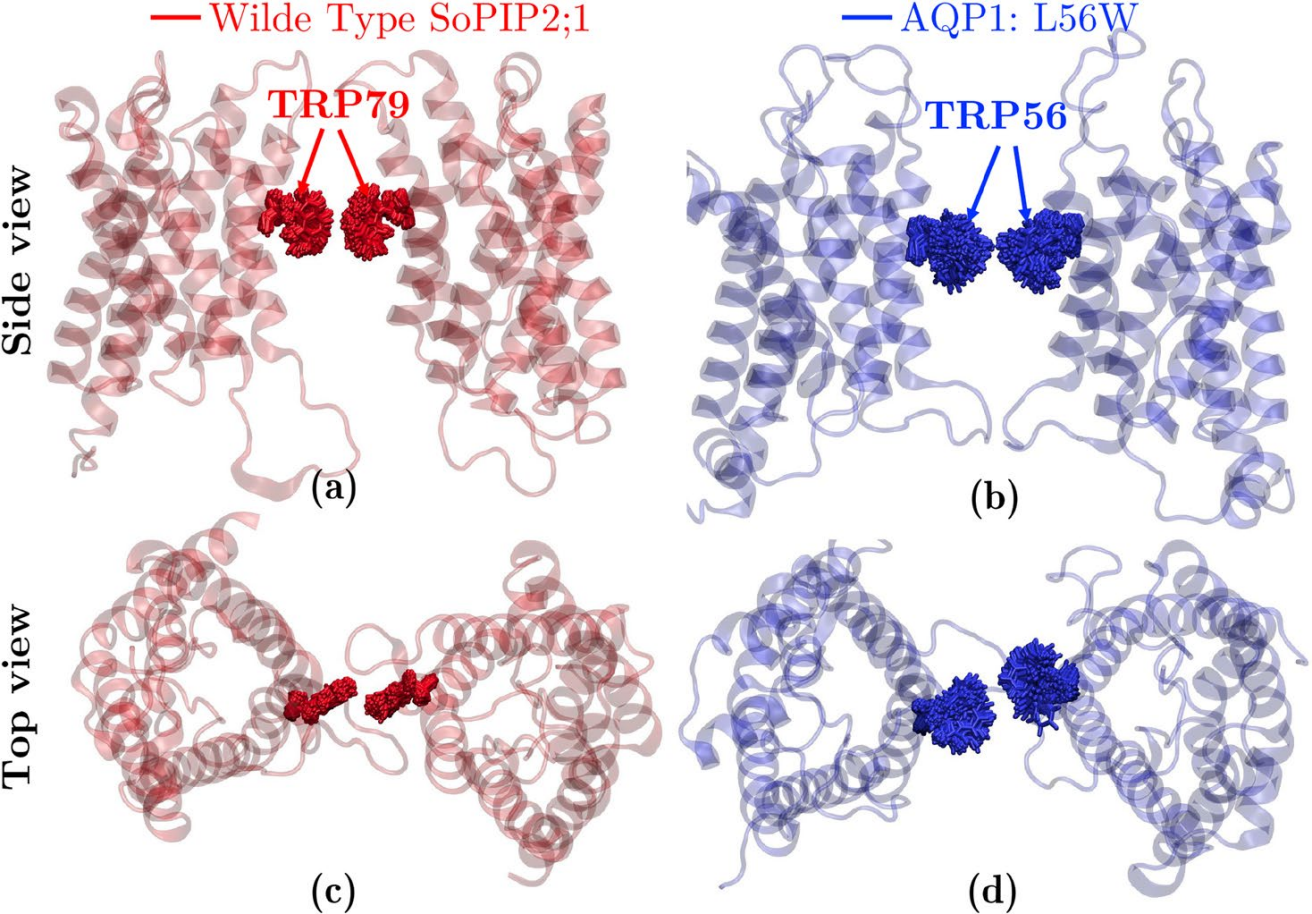
Molecular structure of WT SoPIP2;1 (red; a, c) and mutated bAQP1:L56W (blue; b, d) in ribbon representation. Two monomers are removed to allow the view into the central pore. The sampling motion of native Trp79 of SoPIP2;1 (red) and mutated Trp56 of bAQP (blue) are also shown. The trajectory of the native Trp79 indicates that this residue has limited motion and stays close to its initial configuration during the simulation, however, the mutated Trp56 in bAQP1:L56W moves quite a bit and goes to various positional configurations.

In addition, we compared the trajectories of these two AQPs during 200 ns simulations in Figure 10. As shown in the figure, the motion of the native Trp79 in the plant AQP during the MD simulations suggests that it stays in a stable configuration with limited movement. However, Trp56 in the mutated mammalian bAQP1 undergoes positional changes and adopts multiple configurations throughout the simulation.

**FIGURE 11:**
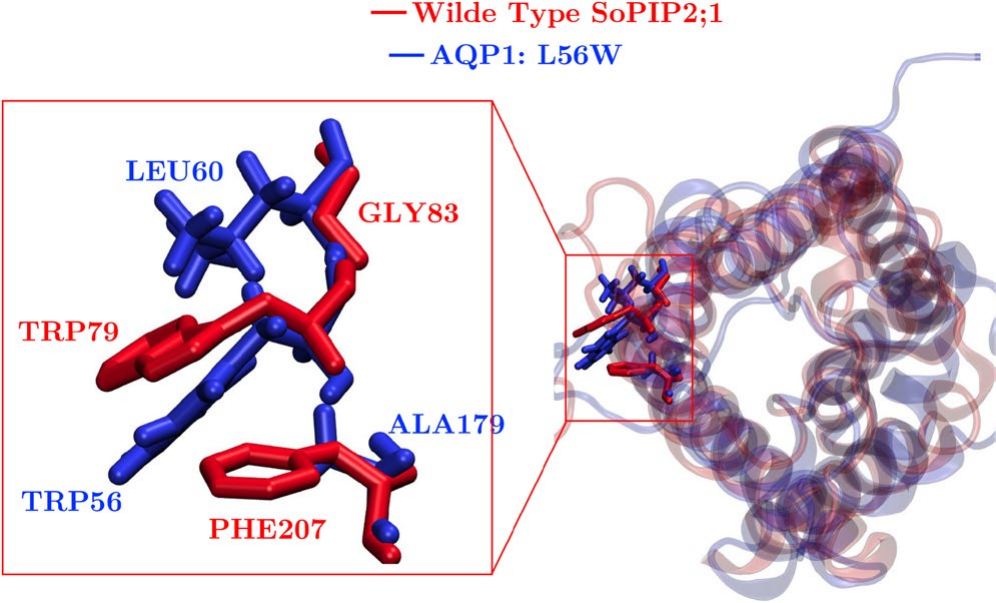
Structural overlay of the WT SoPIP2;1 and the bAQP1:L56W mutant. The close‐up shows the nearby residues to the native Trp79 and mutated Trp56 in WT SoPIP2;1 and mutated bAQP1:L56W, respectively.

A close examination of residues surrounding the Trp79 site in WT SoPIP2;1 and the Trp56 site in bAQP1:L56W mutant explains the observed differences in dynamics (Figure 11). Trp79 of SoPIP2;1 is stabilized by the benzyl ring of Phe207, which maintains an occluded conformation. Sequence comparison between SoPIP2;1 and other PIP2;1 AQPs indicates strong conservation of Phe207. In mammalian and gas‐permeable AQPs, this phenylalanine is replaced by smaller residues such as alanine, glycine, or threonine. For example, in bAQP1, the equivalent position corresponds to Ala179.

**FIGURE 12:**
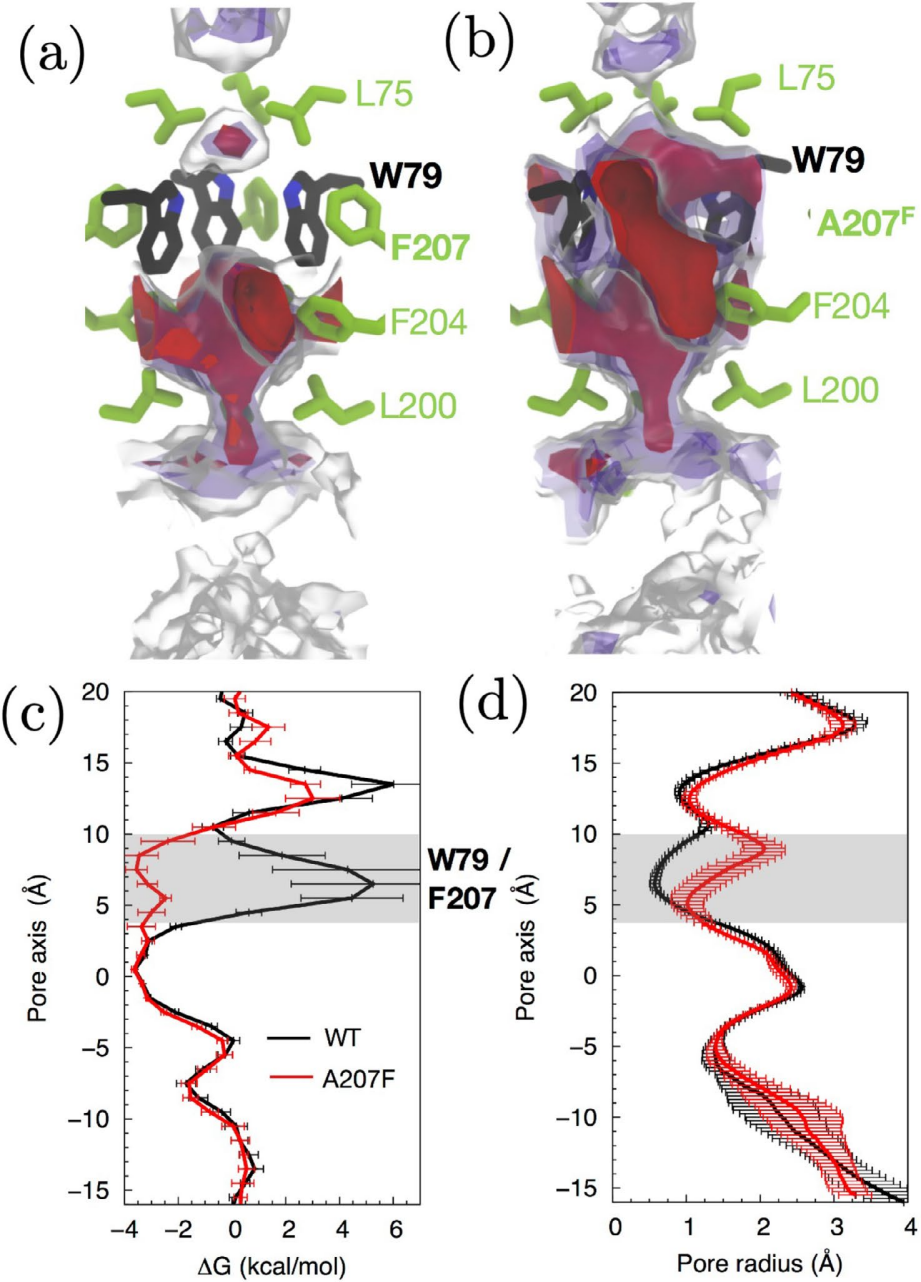
(a) Free energy (ΔG) maps of $O_{2}$ insertion in the central pore of WT SoPIP2;1. (b) ΔG map in the F207A mutant. The red maps correspond to an $O_{2}$ insertion ΔG ($\Delta G_{O_{2}}$) of −2 kcal/mol. The purple maps correspond to a $\Delta G_{O_{2}}$ of 0 kcal/mol. The gray‐transparent maps correspond to a $\Delta G_{O_{2}}$ value of 1.6 kcal/mol. (c) 1D $\Delta G_{O_{2}}$ profiles along the central pore. $\Delta G_{O_{2}}$ barrier against $O_{2}$ associated with Trp79 residues formed in WT, located in between z=4 Å and z=10 Å, was eliminated in the F207A mutant. (d) Hole profiles of the central pore, indicating that Trp79 residues form the narrowest section of the pore. The mutation of Phe207 to Ala removes this constriction. Error bars represent standard deviations across the eight 10‐ns ILS segments (20−30 ns, 30−40 ns, …), as described in Section 2.3.

We hypothesized that mutating Phe207 of SoPIP2;1 to a smaller residue, such as alanine, could increase the conformational flexibility of Trp79 and consequently enhance gas permeability through the central pore. As expected, mutating Phe207 to alanine (F207A) relieved the constriction at the Trp79 site (Figure 12d), resulting in a dramatic reduction of the energy barrier for $O_{2}$ permeation (Figure 12a–c).

**FIGURE 13:**
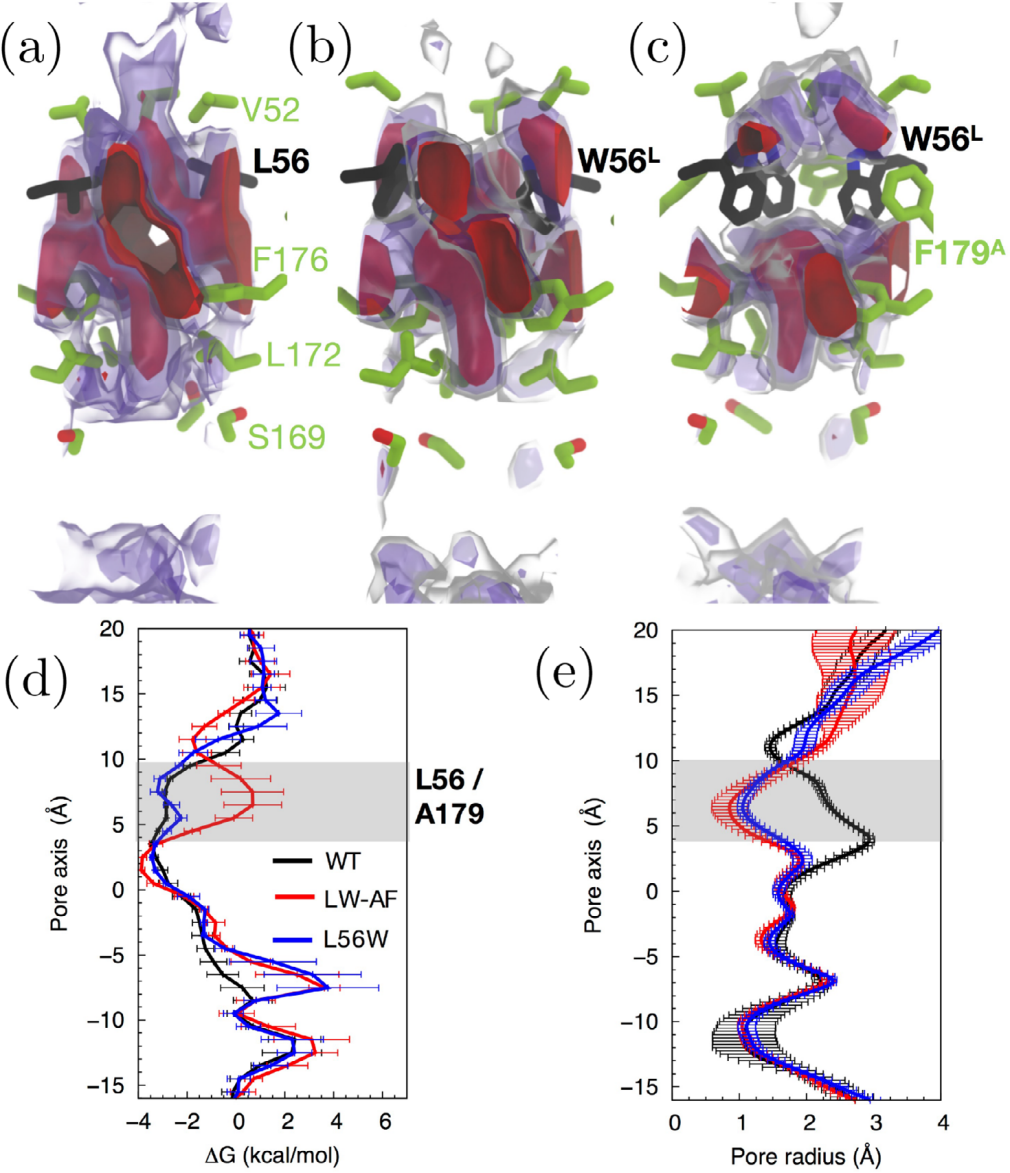
(a–c) ΔG maps of $O_{2}$ insertion in the central pore of WT bAQP1, and its L56W/A179F and L56W mutants. The red maps correspond to a $\Delta G_{O_{2}}$ of −2 kcal/mol. The purple maps correspond to a $\Delta G_{O_{2}}$ of 0 kcal/mol. The gray‐transparent maps correspond to a $\Delta G_{O_{2}}$ of 1.6 kcal/mol. (d) $\Delta G_{O_{2}}$ profiles along the central pore. The L56W/A179F mutant introduced a $\Delta G_{O_{2}}$ barrier between z=4 Å and z=10 Å, equivalent to the Trp79 site in SoPIP2;1. (e) Hole profiles of the central pore. Error bars represent standard deviations across the eight 10‐ns ILS segments (20−30 ns, 30−40 ns, …), as described in Section 2.3.

The free‐energy profiles in Figures 12 and 13 include standard deviations computed from eight independent ILS segments (20−30 ns, 30−40 ns, …), each producing a separate free‐energy estimate. The variation across these segments is shown as error bars. In contrast, the profiles shown earlier in Figures 2, 4, and 8 correspond either to explicit‐sampling results or to ILS profiles averaged over all segments, for which individual segment‐resolved standard deviations are not plotted.

**FIGURE 14:**
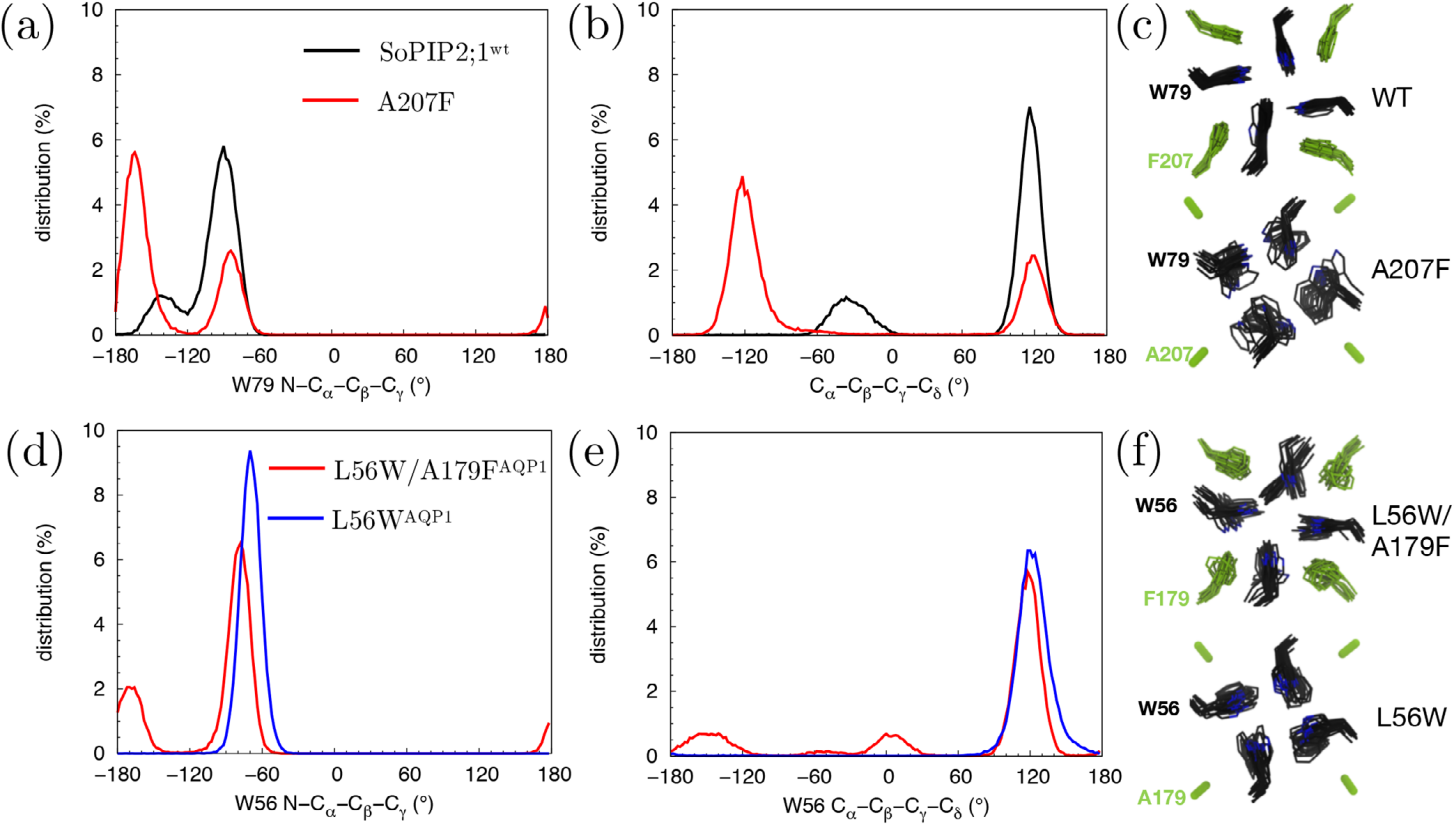
Side chain dynamics of the bottleneck Trp residues of the central pores characterized by their $N-C_{\alpha }-C_{\beta }-C_{\gamma }$ ($\chi_{1}$) and $C_{\alpha }-C_{\beta }-C_{\gamma }-C_{\delta }$ ($\chi_{2}$) dihedral angles. (a, b) $\chi_{1}$ and $\chi_{2}$ of W79 in WT SoPIP2;1 and its A207F mutant. (c) Conformations of W79 and F207 sampled during 100‐ns simulations. In WT, although one of the four Trp79 residues transited from $\chi_{1}/\chi_{2}$ of −90°/120° to −150°/30°, the pore remained occluded. (d–f) Conformations of W56 in L56W/A179F and L56W AQP1 mutants.

In WT SoPIP2;1, most Trp79 conformations cluster around $\chi_{1}/\chi_{2}$ of $-90^{{}^{\circ}}/120^{{}^{\circ}}$ (Figure 14a,b). Although one Trp79 residue deviates to $\chi_{1}/\chi_{2}$ of $-150^{{}^{\circ}}/30^{{}^{\circ}}$ during the simulation, the pore remains occluded (Figure 14c). In contrast, the F207A mutant exhibits a partial opening of the pore as Trp79 transitions to $\chi_{1}/\chi_{2}$ values of approximately $-170^{{}^{\circ}}/-20^{{}^{\circ}}$ (Figure 14a–c), making the central pore more favorable for $O_{2}$ permeation and confirming our hypothesis.

In a reverse engineering approach for bAQP1, we computationally introduced two mutations: L56 to tryptophan and A179 to phenylalanine (L56W/A179F). The L56W/A179F mutant introduces a hindrance against $O_{2}$ diffusion through the central pore, as evidenced by the formation of a Δ *G* barrier for $O_{2}$ at the W56 site (Figure 13a–c). In contrast, no $O_{2}$ blockage is observed in the L56W single mutant. HOLE analysis reveals bottleneck pore radii of ∼0.8 Å for the L56W/A179F mutant and 1.0 Å for the L56W mutant (Figure 13d), indicating a greater degree of pore occlusion in the L56W/A179F double mutant.

## 4 Concluding Remarks

We investigated the gas permeability of Spinach PIP2;1 through its central pore using MD simulations on a tetrameric SoPIP2;1 structure embedded in a lipid bilayer and surrounded by bulk aqueous solution. Various MD approaches were employed to calculate the energetics of $O_{2}$ and ${\mathrm{CO}}_{2}$ partitioning within the central pore, including explicit flooding simulations (ELS), ILS, and US. All three methods yielded consistent free energy profiles for gas permeability via the central pore. ELS results indicate that the central pore of SoPIP2;1 is not readily permeable to gases, with energy barriers of 3.5–3.8 kcal/mol for $O_{2}$ and ${\mathrm{CO}}_{2}$ at the site of the bulky residue Trp76, which introduces a significant constriction along the pore. These findings are confirmed by ILS and US for the energy profiles of $O_{2}$ permeability through the central pore.

Our in silico mutagenesis studies demonstrate that replacing large residues at constriction sites with smaller residues (e.g., alanine) converts the central pore into a gas reservoir that rapidly fills with hydrophobic $O_{2}$ and ${\mathrm{CO}}_{2}$ molecules. In a reverse‐engineering approach inspired by SoPIP2;1 architecture, we designed a double mutant of bAQP1 (L56W/A179F) with a central pore that is effectively impermeable to $O_{2}$ and ${\mathrm{CO}}_{2}$.

These results also help reconcile longstanding discrepancies in the literature regarding AQP‐mediated gas transport. By identifying the pore‐lining residues responsible for major free‐energy barriers, our study provides a structural explanation for why plant PIP2;1 AQPs are gas‐impermeable, whereas mammalian AQP1 exhibits measurable gas permeability. The strong agreement across explicit flooding, implicit sampling, and umbrella‐sampling free‐energy calculations reinforces the robustness of these observations and clarifies how differences in pore architecture across AQP subfamilies contribute to previously conflicting experimental findings.

## Funding

This work was supported by the National Institutes of Health (P41‐GM104601 and R24‐GM145965, U01‐GM111251, and R01‐DK128315), the Office of Naval Research (ONR N00014‐16‐1‐2535), the Myers/Scarpa endowed chair, the National Science Foundation Supercomputing Centers (ACCESS grant number MCA06N060), the National Science Foundation (award OAC 2005572), and the State of Illinois.

## Conflicts of Interest

The authors declare no conflicts of interest.

## Data Availability

All simulation data and analysis files associated with this study, including input files for system construction, equilibrium and production MD simulations, alignment and analysis scripts, as well as all ELS and ILS trajectories for wild‐type and mutant SoPIP2;1 systems, are deposited at Zenodo. To comply with file‐size limits, ELS and ILS datasets are provided as two separate Zenodo repositories. The datasets include structure files, aligned trajectories, configuration files, and all analysis scripts needed to reproduce the free‐energy profiles and figures presented in this manuscript. Zenodo DOIs are: 10.5281/zenodo.19139131 (ELS dataset) and 10.5281/zenodo.19141358 (ILS dataset).
